# A minimal impact of long-term S-flurbiprofen plaster application on kidney function in osteoarthritis patients

**DOI:** 10.1007/s10157-017-1406-9

**Published:** 2017-04-04

**Authors:** Noboru Otsuka, Ikuko Yataba, Isao Matsushita, Hideo Matsumoto, Yuichi Hoshino, Yoshio Terada

**Affiliations:** 1 Development Headquarters, Taisho Pharmaceutical Co., Ltd., 3-24-1 Takada, Toshima-ku, Tokyo, 170-8633 Japan; 2 Institute for Integrated Sports Medicine, Keio University School of Medicine, 35 Shinanomachi, Shinjuku-ku, Tokyo, 160-8582 Japan; 3 Orthopedics Surgery, School of Medicine, Jichi Medical University, 3111-1 Yakushiji, Shimotsuke, Tochigi 329-0498 Japan; 4 Department of Endocrinology, Metabolism and Nephrology, Kochi Medical School, Kochi University, Kohasu, Oko-cho, Nankoku, Kochi 783-8505 Japan

**Keywords:** Acute kidney injury, Drug-induced kidney disease, Kidney function, Long-term safety, Nonsteroidal anti-inflammatory drugs, S-flurbiprofen

## Abstract

**Background:**

The number of kidney injury due to nonsteroidal anti-inflammatory drugs (NSAIDs) is the largest among drug-induced kidney diseases. Newly developed NSAID plaster containing S-flurbiprofen (SFP) shows innovative percutaneous absorption. However, systemic exposure to SFP following the repeated application of 80 mg/day was estimated as comparable to that of oral 120 mg/day flurbiprofen and prolonged use of topical NSAIDs is common in clinical practice. Thus, we report the safety focusing on the kidney function after long-term application of SFP plaster (SFPP).

**Methods:**

A total of 201 osteoarthritis patients (mean age; 66.3, 151 females, mean estimated glomerular filtration rate; 74.6 mL/min/1.73 mm^2^) were applied 40 or 80 mg SFPP for 52 weeks, and kidney function was examined by blood urea nitrogen (BUN), serum creatinine (SCr), eGFR, and urinalysis.

**Results:**

161 (80.1%) patients completed 52-week application. In both groups of 40 and 80 mg, small but statistically significant increases were observed in BUN (mean 1.91 and 1.89 mg/dL, *p* < 0.05) and SCr (mean 0.019 and 0.022 mg/dL, *p* < 0.05). Although abnormal changes in laboratory test for renal function were observed in seven patients, all the changes were small and subclinical. Acute kidney injury was observed in two patients. Meanwhile, the investigators denied the relevance of SFPP according to the clinical course.

**Conclusion:**

Toward the end of 52-week application, a statistically significant increase in SCr was observed in both 40 and 80 mg, but increment was small and subclinical. Attention should be paid to kidney function when applying SFPP to patients with multiple risk factors.

## Introduction

Although nonsteroidal ant-inflammatory drugs (NSAIDs) have been widely used to treat the musculoskeletal diseases, those accompany several safety concerns [1]. Among the concerns, prevalence of kidney injury due to NSAIDs is relatively low, while its impact on prognosis of the patients is significant [2]. The number of patients who developed drug-induced kidney disease (DIKD) due to NSAIDs is the largest among DIKD patients because of common and wide use [2, 3]. Cases of DIKD have been reported not only with oral NSAIDs but topical NSAIDs [4].

Topical NSAIDs are used more commonly than oral NSAIDs mainly because of its reduced adverse effect on gastrointestinal tract, whereas percutaneous absorption and penetration into deep tissue is still insufficient to achieve a satisfactory effect [5, 6]. To bring one solution to the problem, we selected S-flurbiprofen (SFP) from its strong inhibition of cyclooxygenase and good skin permeability [7–9], and developed tape-type patch (SFP plaster: SFPP) with dramatically improved percutaneous absorption and penetration into deep tissue [7, 10].

SFPP showed statistically significant efficacy compared with placebo and flurbiprofen (FP) patch, which is available for the treatment of osteoarthritis, and had an acceptable safety profile [11, 12]. On the other hand, systemic exposure to SFP following the repeated application of 80 mg/day (SFPP 40 mg × 2 patches/day) for 7 days was estimated to be comparable to that of oral formulations of FP at a usual dosage (40 mg × 3/day) [13]. In clinical practice, topical NSAIDs are usually used for long-term treatment, so SFPP is expected to be used for long periods. It is well known that NSAIDs induce kidney injury [14, 15], but we can find no prospective studies investigating long-term effect on kidney function of topical NSAIDs. We conducted a 52-week study of SFPP at dosages up to 80 mg/day, and here, we report the safety focusing on the kidney function.

## Materials and methods

### Patients, drug disposition, and study protocol

This was a multi-center, open-label, uncontrolled prospective study conducted at 11 study sites in Japan. All the investigators were orthopedist. SFPP is a 10 cm × 14 cm, tape-type patch containing 40 mg of SFP per patch (Tokuhon Corporation, Tokyo Japan). Detailed protocol was described in our previous report [16].

In this study, osteoarthritis (OA) patients who had been treated with existing NSAIDs were enrolled and treated with the same NSAIDs for 2 weeks as an observation period. After the observation period, either SFPP 40 mg (40 mg × 1 patch, 101 patients) or 80 mg (40 mg × 2 patches, 100 patients) was applied to these patients instead of previous NSAIDs for 52 weeks. The sites of SFPP application were knee, lumber spine, cervical spine, and other sites, and detail was described in the previous report [16]. Patient demographics data (gender, age, weight, body mass index, blood urea nitrogen, serum creatinine, and estimated glomerular filtration rate) were collected.

In all the patients, plasma concentration of SFP was measured at 4, 8, and 12 weeks after the SFPP application using high-performance liquid chromatography [10] at Sumika Chemical Analysis Service, Ltd. (Osaka, Japan) to evaluate the systemic exposure to SFP.

Serum creatinine (SCr), blood urea nitrogen (BUN), and urinalysis results were examined at 2 and 4 weeks after the SFPP application and thereafter at 4-week intervals until 52 weeks. From SCr, age, and body height and weight, mean estimated glomerular filtration rate (eGFR) was calculated. To examine renal function in detail, time courses of the mean values for SCr, BUN, and eGFR, stratified according to the patients’ eGFR (30–59, 60–89, 90–) at baseline, and eGFR stratified according to the patients’ age (–64, 65–74, 75–), were analyzed. Medical interview on the patient’s condition was carried out at every visit, and overall results were evaluated from both the laboratory data and the answer of the interview. In addition, study results from the patients who showed distinguished decrease in eGFR after SFPP application were analyzed including the risk factors (complications and concomitant drugs), and the relevance to SFPP was assessed.

### Statistical analysis

All analyses were carried out according to the prespecified statistical analysis plan using SAS^®^ 9.2. Continuous outcomes in the laboratory tests were analyzed using a paired t test. The significance level was set at 5% (two-sided).

## Results

In patient demographics, 59 patients (29.4%) aged 75 or more, maximum BUN was 25.3 mg/dL, and minimum eGFR was 43 mL/min/1.73 m^2^ (Table 1).

**Table 1:** Patient demographics and baseline of the laboratory test for kidney function

	40 mg (1 patch)	80 mg (2 patches)	Total
	*N* = 101	*N* = 100	*N* = 201
Gender			
Female	72 (71.3)	79 (79.0)	151(75.1)
Age (years)			
Range	33–87	30–85	30–87
–64	36 (35.6)	38 (38.0)	74 (36.8)
65–74	35 (34.7)	33 (33.0)	68 (33.8)
75–	30 (29.7)	29 (29.0)	59 (29.4)
Mean ± SD	66.2 ± 12.1	66.4 ± 11.5	66.3 ± 11.8
Weight (kg)			
Range	34.1–102.8	40.2–96.3	34.1–102.8
Mean ± SD	59.1 ± 10.5	61.8 ± 12.4	60.5 ± 11.6
BMI (kg/m^2^)			
Range	14.6–32.7	17.3–42.8	14.6–42.8
Mean ± SD	24.2 ± 3.1	25.3 ± 4.5	24.8 ± 3.9
BUN (mg/dL)			
Range	7.2–24.9	8.2–25.3	7.2–25.3
Mean ± SD	15.1 ± 3.6	16.1 ± 3.7	15.6 ± 3.7
Serum creatinine (mg/dL)			
Range	0.43–1.11	0.38–1.10	0.38–1.11
Mean ± SD	0.68 ± 0.16	0.68 ± 0.13	0.68 ± 0.15
eGFR (mL/min/1.73 m^2^)			
Range	43–109	48–141	43–141
Mean ± SD	75.7 ± 14.6	73.6 ± 16.4	74.6 ± 15.5

(): %

Number of the patients who completed 52-week application was 82 for 40 mg and 79 for 80 mg. More than 90% of the patients achieved 80%-adherence in both 40 and 80 mg groups (Table 2).

**Table 2:** Completion of 52 weeks and achievement of 80% adherence

	40 mg (1 patch)	80 mg (2 patches)	Total
	*N* = 101	*N* = 100	*N* = 201
Completed 52 weeks	82 (81.2)	79 (79.0)	161 (80.1)
Achieved 80% adherence	93 (92.1)	93 (93.0)	186 (92.5)

80% adherence means patients attached ≥80% patches scheduled for each patient

(): %

**Fig. 1:**
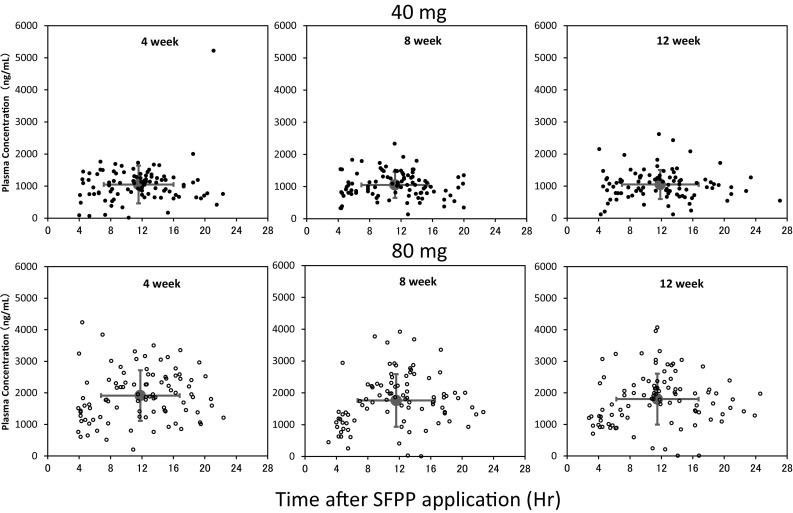
Scatter plots of plasma concentration of SFP vs time after application at 4, 8, and 12 weeks [40 mg (*filled circle*), 80 mg (*white circle*)]. *Red circle* represents the mean of plasma concentration of SFP and application time. *Vertical red line* SD of plasma concentration of SFP. *Horizontal red line* SD of application time

Mean plasma SFP concentration after 80 mg application was twice as high as 40 mg and each dose showed constant plasma concentration throughout the examined period from 4 to 12weeks (Fig. 1). No difference in plasma concentration was observed among the application sites (data not shown). One patient in 40 mg showed abnormally high value (5220 ng/mL) at 4 weeks, and normal values at 8 weeks (720 ng/mL) and 12 weeks (1030 ng/mL). This patient took neither FP preparation nor drugs with inhibition of CYP2C9, which is a key metabolic enzyme of SFP [17].

**Fig. 2:**
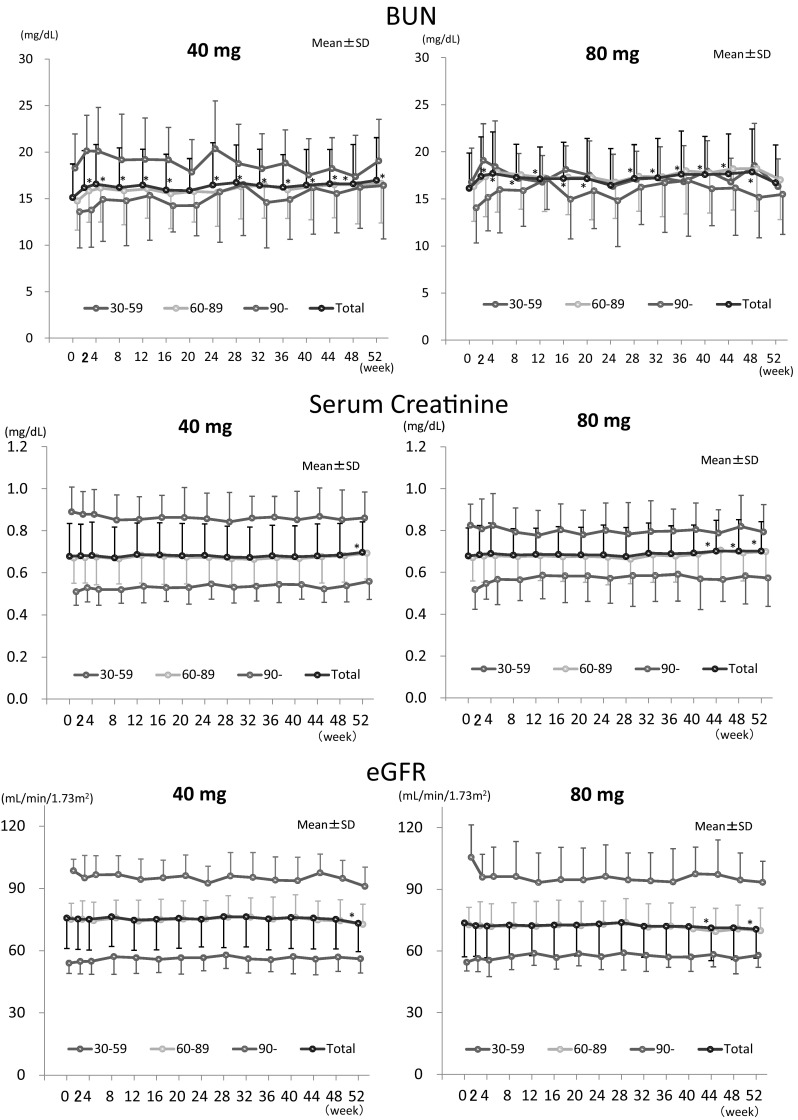
Time course of laboratory tests for kidney function. Data are means ± SD. Numbers of patients in 40 mg are 16 (0 week) and 13 (52 weeks) for eGFR (mL/min/1.73 m^2^) 30–59, 68 (0 week) and 54 (52 weeks) for 60–89, 17 (0 week) and 14 (52 weeks) for 90-, and 101 (0 week) and 81 (52 weeks) for total. Numbers of patients in 80 mg are 17 (0-week) and 15 (52-week) for eGFR (mL/min/1.73 m^2^) 30–59, 71 (0-week) and 54 (52-week) for 60–89, 12 (0-week) and 10 (52-week) for 90-, and 100 (0-week) and 79 (52-week) for Total. *p < 0.05 vs baseline (0w) of total. BUN and Serum Creatinine data for total were published in the previous report [16]

**Fig. 3:**
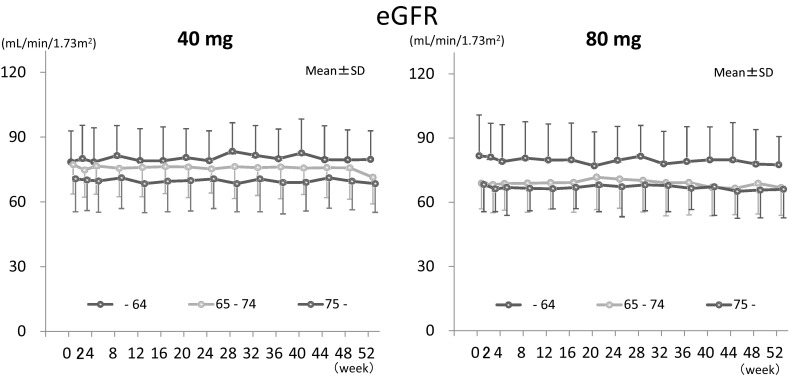
Time course of eGFR stratified according to the age of the patients at baseline. Data are means ± SD. Numbers of patients in 40 mg are 36 (0 week) and 28 (52 weeks) for aged –64 years, 35 (0-week) and 27 (52-week) for 65–74, and 30 (0 week) and 26 (52 weeks) for 75– and in 80 mg are 38 (0 week) and 30 (52 weeks) for aged –64 years, 33 (0 week) and 25 (52 weeks) for 65–74, and 29 (0 week) and 24 (52 weeks) for 75–

Time courses of laboratory tests for kidney function are shown in Figs. 2 and 3. Time courses of BUN and SCr in total patients were already described in our previous report [16]. For eGFR in total, decreases were small, although they were statistically significant at 52 weeks in 40 mg and at 44 and 52 weeks in 80 mg (maximum change of 40 and 80 mg were 2.9 and 2.8 mL/min/1.73 m^2^) (Fig. 2). From stratified analysis in patients who showed low eGFR (30–60) at the baseline, changes (mean ± SD of 40, 80 mg) at 52 weeks were: BUN: 0.91 ± 2.40, −0.58 ± 3.62 mg/dL, SCr: −0.024 ± 0.064, −0.045 ± 0.053 mg/dL, and eGFR: 1.7 ± 4.5, 3.8 ± 4.3 mL/min/1.73 m^2^. There were no relations between the changes in BUN, SCr and eGFR, and application period or dosage (Fig. 2). From stratified analysis according to age (–64, 65–74, 75–), there was no relation observed between the changes in eGFR and age (Fig. 3). Abnormal changes in laboratory test for renal function are listed in Table 3. Investigators did not deny those relevancies to SFPP. All the changes were subclinical.

**Table 3:** Number of patients who showed abnormal changes in laboratory test for kidney function

Patients/total (%)	40 mg (1 patch)		80 mg (2 patches)	
	2/101 (2.0)		5/100 (5.0)	
	Case (%)	Baseline/max	Case (%)	Baseline/max
Blood urea nitrogen ↑	1 (1.0)	15.1/24.7	3 (3.0)	15.0/24.3
				21.1/31.3
				16.6/24.5
Serum creatinine↑	1 (1.0)	0.70/0.93*		
Hematuria (+)	1 (1.0)	±/2+*	2 (2.0)	−/2+**
				−/2+
Proteinuria (+)			1 (1.0)	−/+**

Asterisks * and ** mean abnormal changes seen in the same patient, respectively

Max means the maximum value among abnormal changes. Blood urea nitrogen; mg/dL, Serum creatinine; mg/dL

**Fig. 4:**
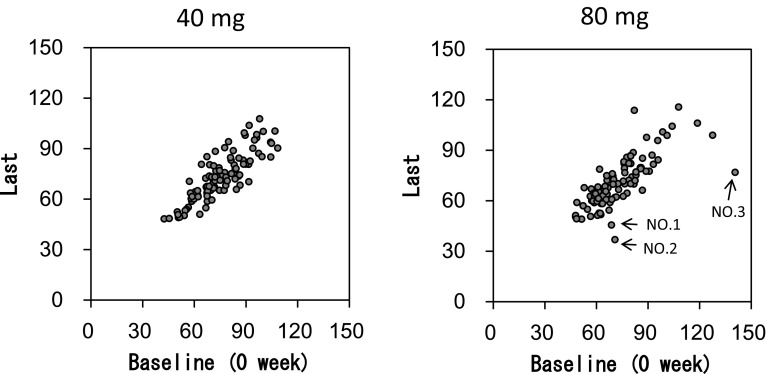
Scatter plots of eGFR (mL/min/1.73 m^2^) at baseline vs last. Decrease at last was observed in three patients attached 80 mg (indicated by →). Acute kidney injury was caused by pyelonephritis in the first patient (No. 1), and dehydration accompanied by atrial fibrillation in the second patient (No. 2). The third patient (No. 3) showed higher eGFR at baseline than other time points. Except for the baseline, consistent eGFR was confirmed both in observation and application period. It was not considered as AKI could be due to the artifact of SCr measurement (No. 3)

Remarkable decreases in eGFR at the end of the application period (Last) were observed in three patients whom 80 mg SFPP was applied (Fig. 4). Among three patients, two patients (Nos. 1 and 3) completed 52-week application and one patient (No. 2) discontinued application at 24 weeks due to atrial fibrillation. The first patient (age: 75, female, No. 1) showed decrease in eGFR (68.8 → 39.0) at 44 weeks and it was considered as acute kidney injury (AKI) due to pyelonephritis. This patient showed fever, malaise, anorexia, and dysuria. Gram-negative bacillus (3+) and leukocyte (3+) were detected from urine sample, and E. coli was isolated from urine culture. After 9 days of hospitalization, symptoms were improved. SFPP administration was continued during hospitalization. Outpatients care was continued and pyelonephritis was disappeared 22 days after onset and eGFR returned slowly and gradually (39.0→45.5, at 52 weeks) under continuous treatment with SFPP after the improvement of pyelonephritis.

The second patient (age: 77, Female, no. 2) showed decrease in eGFR (70.8 → 36.8) at 24-week and it was considered as AKI due to dehydration accompanied by atrial fibrillation (AF). Rapid recovery (36.8 →52.6) from AKI was observed after disappearance of AF.

Investigators denied the causal relationship between AKI and SFPP of the two cases. Nephrologist and cardiologist agreed with the decision of the investigators.

The third patient (age: 43, male, No. 3) showed higher eGFR (140.7) at baseline than any other time points (eGFR at observation period, 2 and 52 weeks after application: 76.8, 83.0, and 76.8). The high value of eGFR was due to the temporally low value of SCr (0.5 mg/dL).

Among three patients mentioned above, one patient (No. 2) had risk factors, complication of hypertension, and administration of calcium channel blocker. None of three patients had other risk factor including complication of diabetes mellitus and concomitant use of renin-angiotensin inhibitor.

## Discussion

Prevalence of OA increases with aging [18, 19]. In the aging society, a large number of patients are affected with OA [18]. Especially, knee OA is very common among the elderly women, and it causes remarkable loss in locomotive abilty and impairs their quality of life. NSAIDs have widely been used for a long time in the treatment of OA. Nowadays, topical NSAIDs are highly recommended than oreal NSAIDs for the treatment of knee OA [5] and more potent topical NSAIDs with an acceptable safety profile is highly expected.

We have developed SFPP, a tape-type patch of SFP (an active form of FP), showing dramatically improved transdermal absorption. It was reported that oral FP at daily dosage of 200 mg (50 mg × 4/day) for 4 weeks cause no overall chronic adverse effects on renal function in patients with moderate renal insufficiency [20]. However, there have been no reports of the long-term effects of oral FP on kidney function.

In this study, from the plasma concentration of SFP measured at 4, 8, and 12 weeks after SFPP application, consistent systemic exposure was comfirmed in 40 and 80 mg (Fig. 1). Furthermore, excellent adherence was kept throughout this study (Table 2), and kidney function was evaluated under the consistent systemic exposure to SFP, as a consequence.

In our previous studies, small but staistically significant increase in BUN was obsereved with 2-week application of SFPP [11, 12], bringing concerns that further increase in BUN might be seen with the long-term application. In this study, statistically significant increase was also observed from 2 weeks in 40 and 80 mg, but increments were small and neither further increment nor significant difference between two dosage groups were observed. Increase in BUN due to NSAIDs is well known [21], but detailed time course has not been studied in detail, yet. In case of SFPP, the reason for this paticular time course of increase in BUN is not clear. Toward the end of 52-week application, a statistically significant but small increase in SCr was also observed [16].

It has been reported that CKD is a risk factor for AKI, and the incidence of AKI is associated with pre-existing reduced kidney function [22]. In stratified analysis by baseline eGFR, even the lowest eGFR (30–59 mL/min/1.73 m^2^) group did not show apparent change in kidney function in this study (Fig. 2). Older age is also listed as the risk factor for drug-induced impairment of renal function [23], but no relation between the changes in eGFR and age was observed in stratified analysis according to the age in both dosage groups.

Abnormal changes in laboratory test for renal function were observed in BUN, SCr, and urinalysis (Table 3), three cases in two patients of 40 mg group and six cases in five patients of 80 mg group. The incidence of abnormal changes seemed to be rather higher in 80 mg group, but all the changes were small and subclinical. We consider that these changes did not mean the DIKD due to SFPP.

In this study, AKI was observed in two patients with high eGFR (≥60 mL/min/1.73 m^2^). One case (Patient No. 1) was due to pyelonephritis and eGFR returned slowly and gradually under continuous treatment with SFPP after the improvement of pyelonephritis. There are two case reports describing acute interstitial nephritis induced by oral FP [24, 25]. In both cases, after withdrawal of FP, steroid therapy could not demonstrate that sufficient recovery [24, 25]. It is not clear if continuous use of SFPP influenced the recovery or not. Another case (Patient No. 2) was considered to be due to dehydration accompanied by atrial fibrillation. It is known that natriuretic peptides secreted from cardiomyocytes cause dehydration [26, 27]. Furthermore, the patient complained that she could not drink or eat. We have concluded that the AKI was very likely caused by dehydration. Rapid recovery was observed after disappearance of AF. There is one case report describing acute renal insufficiency after oral FP with angiotensin converting enzyme inhibitor [28]. Influence of the risk factors (hypertension and its treatment) of this patients (No. 2) was not clear. There were no other patients who showed AKI.

No reports are available on the relative risk of FP causing AKI. In a systematic review and meta-analysis of observational studies, AKI risk of nine NSAIDs was investigated and statistical difference was not found [29]. A retrospective study using the Taiwanese National Health Insurance database has shown that use of NSAIDs is a significant risk factor for dialysis commencement and the odds ratio of propionate delivatives, to which SFP is belonging, is relatively low [30]. Our result was consistent with this report.

The incidence rate of CKD increases with aging [15], and NSAID is one of the major causes of DIKD [15, 31]. The guideline for the diagnosis and treatment of CKD published by KDIGO (Kidney disease improving global outcomes) does not recommend prolonged use of NSAIDs in patients with eGFR <60 mL/min/1.73 m^2^ or use in patients with eGFR <60 mL/min/1.73 m^2^ and severe complications [32]. From our result that no apparent kidney functin loss were observed even in the lowest eGFR (30–59 mL/min/1.73 m^2^) patients in 80 mg group, SFPP seemed to be much safer than existing NSAIDs in the previous report [30].

The limitations of this study are limited number of available data, lack of data from longer application than 52 weeks and data from patients with multiple risk factors for DIKD, and lack of the urinally marker data detecting early damage of interstitume such as β2-microglobulin, and α1-microglobulin. In consideration of limitations, slight but statistically significant decrease of eGFR in the end of the study may have an important meaning. Therefore, attention should be paid to kidney function when SFPP is used in consideration of the limitations listed above. Accumulation of data is needed to conclude the impact of SFPP for long-term application on the kidney function.
